# Legacies of millennial-scale climate oscillations in contemporary biodiversity in eastern North America

**DOI:** 10.1098/rstb.2023.0012

**Published:** 2024-04-08

**Authors:** David Fastovich, Volker C. Radeloff, Benjamin Zuckerberg, John W. Williams

**Affiliations:** ^1^ Department of Geography, University of Wisconsin–Madison, 550 North Park Street, Madison, WI 53706, USA; ^2^ Center for Climatic Research, University of Wisconsin–Madison, 550 North Park Street, Madison, WI 53706, USA; ^3^ Department of Earth and Environmental Sciences, Syracuse University, 141 Crouse Drive, Syracuse, NY 13210, USA; ^4^ SILVIS Laboratory, Department of Forest and Wildlife Ecology, University of Wisconsin–Madison, 1630 Linden Drive, Madison, WI 53706, USA; ^5^ Department of Forest and Wildlife Ecology, University of Wisconsin–Madison, 1630 Linden Drive, Madison, WI 53706, USA

**Keywords:** biodiversity, millennial-scale climate variation, last deglaciation, climate model, climate stability, climate refugia

## Abstract

The Atlantic meridional overturning circulation (AMOC) has caused significant climate changes over the past 90 000 years. Prior work has hypothesized that these millennial-scale climate variations effected past and contemporary biodiversity, but the effects are understudied. Moreover, few biogeographic models have accounted for uncertainties in palaeoclimatic simulations of millennial-scale variability. We examine whether refuges from millennial-scale climate oscillations have left detectable legacies in the patterns of contemporary species richness in eastern North America. We analyse 13 palaeoclimate estimates from climate simulations and proxy-based reconstructions as predictors for the contemporary richness of amphibians, passerine birds, mammals, reptiles and trees. Results suggest that past climate changes owing to AMOC variations have left weak but detectable imprints on the contemporary richness of mammals and trees. High temperature stability, precipitation increase, and an apparent climate fulcrum in the southeastern United States across millennial-scale climate oscillations aligns with high biodiversity in the region. These findings support the hypothesis that the southeastern United States may have acted as a biodiversity refuge. However, for some taxa, the strength and direction of palaeoclimate-richness relationships varies among different palaeoclimate estimates, pointing to the importance of palaeoclimatic ensembles and the need for caution when basing biogeographic interpretations on individual palaeoclimate simulations.

This article is part of the theme issue ‘Ecological novelty and planetary stewardship: biodiversity dynamics in a transforming biosphere’.

## 1. Introduction

Full-glacial climates exert detectable legacies on the contemporary distribution of flora and fauna, both globally and for well-studied regions such as Western Europe [1–7]. The role of past millennial-scale climate variability, however, remains unclear, although several papers have hypothesized that past millennial-scale variability may have been a primary factor governing the current composition and structure of terrestrial ecosystems [8,9]. Large and abrupt climate changes, which were prominent over the last 90 000 years, included local temperature increases of up to 9°C within a few decades, lasted for millennia [10–16], and had global impacts on temperature and precipitation [15,17–21]. Tree species distributions shifted in response to the most recent of these millennial-scale climate oscillations, the Younger Dryas [22,23], but community-climate mismatches were highest during this time period, perhaps from biotic lags to rapidly changing climates [24]. Global-scale analyses of climate stability over the past 21 000 years, a time-period that includes both orbital- and millennial-scale climate variations, shows that contemporary species richness for mammals, birds and amphibians is higher in regions where temperature was more stable and precipitation was more unstable [1].

Several lines of evidence thus provide a basis for hypothesizing that past millennial-scale climate oscillations may have left legacies on contemporary patterns of species richness. To explore this hypothesis, we examined eastern North America as our study system and, in particular, the southeastern United States, which today is a biodiversity hotspot with high species richness for amphibians, birds and trees [25]. This work builds on recent proxy and palaeoclimate model studies of past temperature and precipitation changes in eastern North America for the Younger Dryas, which established spatial patterns and tested hypotheses about the underlying atmospheric mechanisms [26,27]. Notably, these reconstructions showed that the southeastern United States experienced little temperature changes during the Younger Dryas, the most recent millennial-scale climate event. Here, we assess whether reduced millennial-scale climate change in the southeastern United States allowed this region to serve as a refugium for biodiversity, explaining its high amphibian, avian and tree species richness.

All assessments of the effects of past climate changes on modern biodiversity and endemism rely upon palaeoclimatic simulations from climate models [1,3,28,29]. Climate models provide a physics-based representation of past climate dynamics and the necessary climate variables for species and biodiversity modelling. However, past climate simulations have uncertainties caused by climate model parametrization and the uncertain forcings needed to drive simulations. Hence, any climate simulation will differ somewhat from the actual past state of the climate system, with the directionality and magnitude of differences varying among climate models, making model–model and model–data comparisons valuable [30–37]. Uncertainties for simulations of millennial-scale climate variability that are forced by pulses of meltwater into the North Atlantic are particularly high, because these are highly nonlinear events and models differ in their sensitivity to meltwater forcing, through metrics such as magnitude of Atlantic meridional overturning circulation (AMOC) slowdown [38,39]. Reduction in AMOC, in turn, reduces northward heat transport by the Atlantic Ocean and governs the spatial patterns of temperature and precipitation change in regions adjacent to the north Atlantic [40] and across the Northern Hemisphere [41]. That is one reason why climate models continue to show substantial differences in simulated spatial fingerprints at regional to sub-hemispheric scales [38]. For example, in eastern North America, recent multi-model and data-model comparisons [27] documented large discrepancies in simulated precipitation changes following abrupt climate changes forced by pulses of meltwater into the north Atlantic in three atmosphere–ocean general circulation models.

Inaccuracies in climate simulations are a challenge for both global change ecology and biodiversity modelling, and are a top cause of uncertainty in predicting future species distributions, particularly at finer spatial scales [42]. A common recommendation in biodiversity modelling is to employ ensembles of both climate simulations and ensembles of biodiversity models [42,43]. However, predictions of past climate change on past species dynamics and contemporary biodiversity often employ just one or two palaeoclimatic simulations [44–50] and do not consider climate simulation errors or differences among climate simulations [1,29,51–54]. For example, the TraCE-21ka simulations have been particularly heavily relied on for biogeographic modelling [55,56], because they offer estimates for a variety of climate variables through the most recent deglaciation at high temporal resolution, and have been benchmarked against proxy-based climate reconstructions [14,32,57–60]. Further, the TraCE-21ka simulations have been used as the primary basis for statistical climate downscaling [61,62]. Yet, the TraCE-21ka simulations are from a single climate model, and the simulations have known deficiencies such as the mistiming of increases in atmospheric CO_2_ concentrations relative to new proxy estimates [58,63,64].

For this special issue and its exploration of biodiversity and stewardship, we first review for a biogeographic audience the primary drivers and modes of past climate variation across timescales and their implications for past and present biodiversity. We then assess whether legacies of past millennial-scale climate changes are detectable in the modern distribution of amphibians, passerine birds, mammals, reptiles and trees in eastern North America. We further assess which modelled palaeoclimate–species relationships are consistently detected across the ensemble of palaeoclimatic simulations that we analyse, and which relationships cannot be confidently assessed owing to intermodel climate simulation differences. To do so, we test whether estimates of past climate change from proxies and a suite of 12 climate simulations are predictors of contemporary biodiversity. Our proxy reconstructions focus on the Younger Dryas (*ca* 12 900–11 700 years before present), the most recent millennial-scale climate event commonly attributed to an input of freshwater into the north Atlantic [65,66] or Arctic Ocean [67]. The Younger Dryas is well documented by proxy data networks and is a common focus for palaeoclimatic simulations, as a representative of other millennial-scale climate events that have occurred over the last 60 000 [68]. We focus on eastern North America and the Younger Dryas to build upon prior proxy data syntheses and data-model comparisons, which suggest that the southeastern United States may have been an area of relative thermal stability during the Younger Dryas [26,27]. The climate simulations that we use include the TraCE-21ka simulations [58,59], a statistically downscaled variant of TraCE-21ka [61] and 10 atmosphere–ocean general circulation model simulations with freshwater forcing that mimic the onset of a millennial-scale climate event [27,38]. We fit spatial error models (SEMs) [69] to identify the individual effects of palaeotemperature, palaeoprecipitation, modern temperature and modern precipitation on contemporary species richness, and assess the strengths of these effects for the different taxonomic groups and palaeoclimatic sources.

## 2. Climate variations at orbital and millennial timescales: processes, patterns and implications for contemporary biodiversity

Over the last 5 million years, the repeated transitions from cold, glacial climates to warm, interglacial climates are closely associated with global changes in ice volume and greenhouse gas concentrations [70]. These changes in ice volume and climate are paced by variations in insolation, altering Earth's energy budget and causing global cooling or warming, thereby initiating glaciation or deglaciation, respectively [71]. Orbitally driven transitions between glacial and interglacial periods can be modelled using orbital geometries for the last 50 million years [72–74]. Climate models of varying complexity can simulate the climates of the Last Glacial Maximum in response to changes in orbital parameters well [33], owing to smooth forcings and quasi-linear responses of the climate system to precession and obliquity [75]. Even though the 100 000 year glacial cycles of the Late Pleistocene are attributed to feedbacks and nonlinearities in the atmosphere–ocean–cryosphere system [76–79], climate models still accurately simulate glaciation in response to eccentricity changes [80].

Glacial–interglacial climate variations have left clear imprints on global contemporary biodiversity. Escape from glacial climate variability can explain high species richness and endemism in the tropics, low biodiversity at high latitudes, and patterns of amphibian, avian, mammalian, floral and tree diversity globally [7,51,81–87]. Climate models that include changes to orbital parameters allow direct assessment of these relationships and broadly support the importance of temperature stability [3,7,51] and, to a lesser extent, precipitation instability [1]. Palaeoclimate simulations from climate models also support both the importance of climate stability and the hypothesis that effective speciation rates were lower in the high latitudes owing to climate-induced extirpations of isolated populations necessary for allopatric speciation [81,88]. For example, temperature velocity after the most recent deglaciation predicts contemporary endemism globally, especially for poorly dispersing amphibians [3]. Accordingly, contemporary species distributions may not be in equilibrium with a contemporary climate [5,6]. Beyond the most recent deglaciation, model predictions of the history of life for mammals and birds that include evolutionary and ecological processes over successive glacial and interglacial cycles throughout South America are strikingly similar to modern observed distributions of avian and mammalian diversity [28].

Climate varies across the full continuum of time scales [89], so focusing too much on glacial–interglacial timescales risks excluding the possible influences of other scales of climate variability on contemporary biodiversity. In particular, climate of the last 90 000 years was marked by rapid, global climate changes at millennial time scales [10–13]. Unlike the regular orbital pacing of glacial cycles, millennial-scale climate oscillations occur irregularly with a quasi-periodicity of *ca* 1470 years [90]. Two classes of millennial-scale climate oscillations are particularly prevalent, with hypothesized links between them. Dansgaard-Oeschger events were first identified in the Greenland ice cores [10] as climatic warm events (interstadials) that initiate abruptly and are followed by a gradual cooling to a colder period (stadials), of which 26 have been identified [13]. The causes of Dansgaard-Oeschger events may be either forced or unforced [65,91–96], but clearly involve a change in deep ocean circulation and global heat redistribution [65,92,93,97]. Heinrich events [68] typically occur during the most extreme Dansgaard-Oeschger stadials [98], and are identified as coarse sediments in north Atlantic Ocean cores, probably rafted by icebergs that may indicate a destabilization of Northern Hemisphere ice sheets [99,100]. This outflow of ice into the north Atlantic Ocean during a Heinrich event is hypothesized to place a low-density freshwater cap over the north Atlantic that limits the subduction of surface ocean waters. Such a weakening of the AMOC may be responsible for a hallmark feature of millennial-scale climate events: the ‘bipolar seesaw’, characterized by Northern Hemispheric cooling and Southern Hemispheric warming [101]. Although this conceptualization oversimplifies the regionally variable climate changes within each hemisphere [26,102], the seesaw framework captures the hemispheric-scale reorganization of heat during these millennial-scale events. Millennial-scale climate oscillations are also linked to shifts in the Intertropical Convergence Zone [103,104], changes in the strength of the Asian monsoon [105], shifts in the austral westerlies [18,106], among other climate changes.

Accurate simulations of millennial-scale climate events and their underlying processes remain a frontier in palaeoclimate modelling, which in turn complicates biogeographic interpretations. A standard experimental design, called a ‘hosing’ experiment, is to prescribe the addition of freshwater to the north Atlantic Ocean, to mimic the natural input of freshwater through the destabilization of ice sheets and ice rafting. First, climate models are run to statistical equilibrium, typically focusing on a time period of interest (e.g. Last Glacial Maximum). Then, a freshwater forcing (or equivalent change in surface ocean salinity) is applied that effectively reduces the subduction of surface ocean water and AMOC strength, and the climate model is allowed to reach a new equilibrium. Freshwater hosing produces abrupt climate changes similar to Dansgaard-Oeschger events in climate models because the AMOC is bistable, and because abrupt changes between strong and weak circulation states of the ocean propagate to rapidly change global climate [107–109]. Bistability of the AMOC, and consequently of the global climate, is a central feature of millennial-scale climate oscillations but a challenge for accurate simulations. Climate models vary in their sensitivity to this tipping element, and the application of the same freshwater forcing results in climate changes that differ in magnitude and spatial patterns among climate models [38,39,110]. Uncertainties in the location and amount of freshwater forcing is an additional complication, with the north Atlantic Ocean as a common region of freshwater forcing [39], but meltwater inputs into the Pacific [111], Arctic [112] and Southern Oceans [113] are also plausible. A greater freshwater forcing typically produces larger changes to the AMOC and global climates [38,39], but climate models may not be as sensitive as the real world [114], so many climate model experiments tend to overprescribe freshwater relative to glaciological constraints [115] to produce climate signals commensurate with palaeoclimatic proxies. Despite these challenges, climate models that include hosing experiments can accurately simulate the broad reorganization of global heat, and show widespread cooling throughout the Northern Hemisphere and warming in the Southern Hemisphere [38]. Meltwater forcing experiments can also accurately simulate changes in temperature seasonality in Europe [116], monsoon changes in Asia [16,117,118] and precipitation changes in the western United States [119]. The Younger Dryas is the most recent major meltwater event in the north Atlantic [120], and one of the best-documented, given the general rule that that proxy data networks decline in density further back in time.

Transient climate simulations attempt to simulate climate evolution through time and capture millennial-scale climate oscillations. Unlike hosing experiments that simulate two climatic equilibria, transient climate simulations update climate model forcings as the climate simulation progresses, often informed by proxy evidence. In the TraCE-21ka simulations, climate evolution of the most recent deglaciation was simulated by updating ice sheet topography, palaeogeography, ice sheet meltwater, orbital geometry and greenhouse gases, yielding millennial-scale climate variations observed in the proxy record [58,59]. Within TraCE-21ka, the Younger Dryas is simulated with a freshwater forcing of 20 m of sea-level rise per 1000 years while all other forcings continued to evolve [58]. By design, such simulations allow for interactions and feedbacks among forcings.

## 3. Methods

### (a) Palaeoclimate model simulations

We combine 10 climate simulations of varying resolution and complexity that employ an equilibrium hosing experimental design from eight atmosphere–ocean general circulation models, plus the transient TraCE-21ka simulations [58,59], and the statistically downscaled variant of the TraCE-21ka simulations by Lorenz *et al*. [61], for a total of 12 palaeoclimate climate simulations. For both the equilibrium and transient simulations, we calculate climate anomalies as the values for the experimental, ‘hosed’ state, minus the control, ‘unhosed’ state. For the transient simulations (TraCE-21ka, TraCE-21ka Downscaled), we subtract the values for a 100-year average centred on 11 100 years before present (representing the early Holocene, with minimal meltwater forcing) from a 100-year average centered on 12 300 years before present (representing the Younger Dryas, with high meltwater forcing).

### (b) Proxy palaeoclimate estimates

We use the palaeotemperature and palaeoprecipitation reconstructions for the Younger Dryas from Fastovich *et al*. [26,27], which were developed using fossil-pollen and branched glycerol dialkyl glycerol tetraethers (brGDGTs) from lacustrine sediments at 42 sites. brGDGTs are organic compounds produced by microorganisms ubiquitous in soils and lake sediments [121,122]. Quantitative temperature estimates are produced through modern calibrations of brGDGT abundances in soils [122–124] and lakes [125,126]. These calibrations are then used on abundances of brGDGTs in lacustrine environments to produce quantitative palaeotemperature estimates. Fossil-pollen reconstructions of mean annual temperature and annual precipitation were reconstructed by Fastovich *et al*. [26,27] using three transfer functions (modern analogue technique, weighted averaging, weighted averaging-partial least squares) trained on contemporary pollen abundances [127–130]. Pollen-based precipitation reconstructions have greater inherent uncertainty than temperature reconstructions [131], but comparisons between our fossil-pollen precipitation reconstructions and precipitation-sensitive proxies from throughout eastern North America demonstrate good agreement [27]. As with the TraCE-21ka simulations, all proxy-based climate anomalies are calculated as the Early Holocene (11 100 years before present) ‘unhosed’ state anomalies subtracted from the mid-Younger Dryas (12 300 years before present) ‘hosed’ state.

### (c) Contemporary species richness

We estimate species richness from the International Union for the Conservation of Nature (IUCN) Red List of Threatened species range maps [132], tree species range maps from a digitized version of the Atlas of United States Trees [133] and BirdLife range maps [134] following Radeloff *et al*. [135] (electronic supplementary material, figure S1). These range maps outline species ranges that we use to estimate species richness by rasterizing species range polygons to an equal-area 50 km grid and summing overlapping ranges. During rasterizing, we assume species presence in all grid cells that overlap with the species range [136] and do not employ a threshold of grid cell coverage for a species to be considered present. We follow this procedure for estimates of species richness for birds, terrestrial mammals, amphibians, reptiles and trees. Bird richness estimates are further filtered to represent only passerine species on the list from Sheard *et al*. [137]. We focus on North American passerines because they are highly detectable, vagile, display a wide range of habitat associations and demonstrate strong sensitivity to weather and climate [138].

We perform all analyses on a common 50 km grid using the North America Albers Equal Area projection and bilinearly interpolate all climate simulations to this grid. Because the proxy reconstructions are point estimates, we use universal kriging to interpolate point estimates from the proxy data to the 50 km grid. We use PyKrige [139] for all kriging with parameters from variograms fit to mean annual average temperature from 1951 to 1980 and mean daily precipitation from 1951 to 1980 from the National Centers for Environmental Prediction for Atmospheric Research Reanalysis [140] and the Global Precipitation Climatology Centre datasets [141], respectively. Kriging is an interpolation procedure that produces full spatial fields from point data. Regions where proxy data are sparse (e.g. the southeastern United States) will have higher uncertainties. However, air temperature and precipitation are spatially autocorrelated, which means kriging probably captures the sign of climate changes for the Younger Dryas, despite palaeoclimate data gaps. We perform sensitivity analyses to assess the role of resolution upon our conclusions by replicating our method on a 100 km grid, as recommended by Hurlbert & Jetz [136]. We find that grid cell size has minimal effect on our conclusions, but larger grid cells reduce the significance of the effect of all palaeoclimate and contemporary climate predictors, probably because of fewer grid cells (electronic supplementary material, figures S8–S13).

### (d) Spatial error modelling

**Figure 1.**
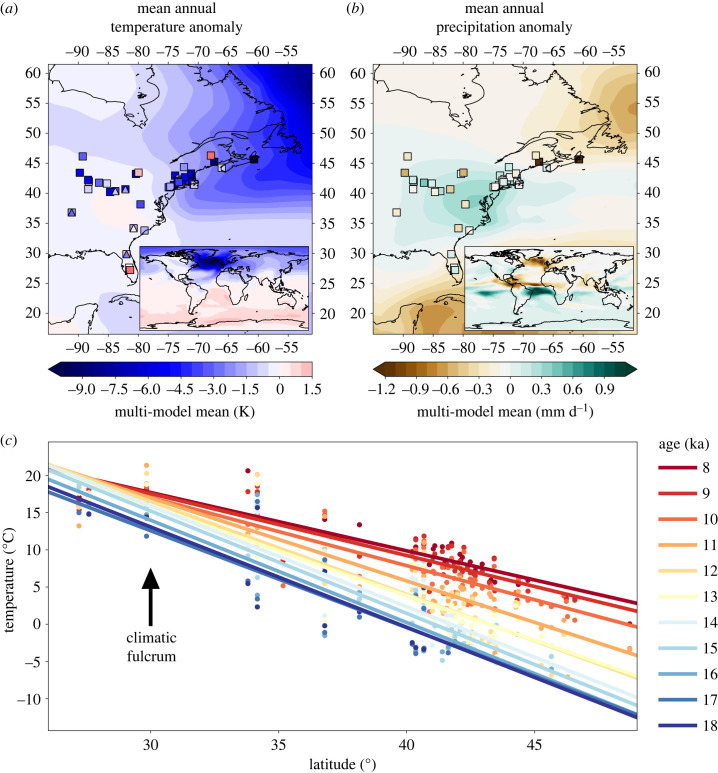
(*a,b*) Hosed–unhosed climate anomalies from 11 climate simulations of a millennial-scale climate event triggered by the input of freshwater into the surface north Atlantic. All climate simulations are averaged to produce a multi-model mean of (*a*) temperature and (*b*) precipitation anomalies, including TraCE-21ka but excluding the downscaled estimates from Lorenz *et al*. [61] for North America. The squares correspond to fossil-pollen based temperature and precipitation reconstructions, and the triangles correspond to branched glycerol tetraether glycerol temperature reconstructions (adapted from Fastovich *et al*. [26,27]). (*c*) Evolution of the latitudinal temperature gradient from 18 to 8 ka based on proxy-based temperature reconstructions, showing an apparent fulcrum at 30° N (indicated by vertical arrow) and the shallowing of the temperature gradient over time, owing to enhanced warming at higher latitudes.

To detect potential legacies of millennial-scale climate events, we build relationships between the palaeoclimate anomalies and contemporary species richness estimates with SEMs (also called spatial autoregressive error models). SEMs calculate a spatial weights matrix and spatial autocorrelation parameters to account for spatial autocorrelation [69,142,143]. Statistical models of this structure have been widely used in biogeography [3,51, e.g.^144^–^148^] to account for spatial autocorrelation [143]. Our analyses are limited to eastern North America defined as east of 92° W and south of 50° N (electronic supplementary material, figure S1). We separately model contemporary richness for amphibians, passerine birds, mammals, reptiles and trees using five predictors: contemporary mean annual temperature, contemporary mean annual precipitation, palaeotemperature anomalies, and palaeoprecipitation anomalies. In the SEMs, palaeoprecipitation-species richness relationships are u-shaped because of a tripole spatial structure (consisting of three spatial loci of opposing sign, i.e. dry in maritime Canada/New England, wet in central eastern North America, and dry in southern Florida) in the precipitation anomalies (figure 1; electronic supplementary material, figure S15), which we address by adding a quadratic term for palaeoprecipitation anomalies. Some palaeotemperature anomalies demonstrate a similar tripole structure, but changes in palaeotemperature are much smaller (*ca* 0.5°C) than palaeoprecipitation which can be as great as 216 mm yr^−1^ for some palaeoprecipitation estimates (figure 1; electronic supplementary material, figures S14 and S15). Hence, we model the influence of palaeotemperature without a quadratic term. Contemporary climate variables are based on 1970–2000 climate means from WorldClim2 [149], reprojected and bilinearly interpolated to the 50 km grid. We fit the SEMs using the *spatialreg* [69] package in R and test various thresholds to construct the spatial weights matrix. Each SEM is fitted with a 50, 100, 200, 400, 600, 800 and 1000 km neighbourhood distances with multiple metrics of model fit informing model selection: log-likelihood, Akaike information criterion (AIC, [150]), Moran's I, variogram and correlogram of the residuals. For all taxonomic groups except passerine birds, the 50 km neighbour threshold provides the best fit, while the 100 km neighbour threshold provides the best fit for passerine birds based on visual inspection, lowest AIC values, residual Moran's I below 0.1, and consistently insignificant variogram and correlograms (electronic supplementary material, table S1). We modelled each taxonomic group separately using our 13 palaeoclimate estimates as predictors, for a total of 65 SEMs.

We examine the relationship among palaeotemperature, palaeoprecipitation, and contemporary biodiversity to test whether the southeastern United States was a biodiversity refuge during the Younger Dryas. We choose these four climate variables to accommodate limits in palaeoclimatic data availability. For 7 of 12 palaeoclimate simulations, only temperature and precipitation anomalies were archived [38]. Additionally, fossil pollen-based proxy reconstructions can only reliably provide estimates for two to three climate dimensions [151] and brGDGTs only reconstruct temperature. Accordingly, we match palaeotemperature and palaeoprecipitation estimates with corresponding contemporary climate parameters. The expectation [1,3,6,51] is that contemporary richness is highest where millennial-scale climate variations were lowest (i.e. where palaeoclimate anomalies are closest to zero). Moreover, given the known sensitivity of deglacial plant distribution dynamics to deglacial temperature changes in mid to high latitudes [152,153], we expect that legacy effects for palaeotemperature are stronger than for palaeoprecipitation. A positive relationship between palaeotemperature and contemporary richness in the SEMs would suggest that the southeastern United States acted as a biodiversity refuge through the Younger Dryas, and perhaps during other millennial-scale climate oscillations. For palaeoprecipitation, we also expect higher contemporary richness in areas of climate stability, modelled here as a convex fit centred on a precipitation anomaly of 0 mm d^−1^. Within this framework, we use an ensemble of 13 palaeoclimate estimates to assess the sensitivity of the fitted relationship to choice of palaeoclimatic model or proxy data. Agreement in the fitted relationship among the SEMs using different palaeoclimate estimates increases confidence in interpretations about the effect of past climate variations on contemporary biodiversity. Conversely, strong qualitative differences among SEMs for some patterns and taxa would suggest that the effects of past climatic variations are too weak to overcome intermodel differences in palaeoclimate simulations.

## 4. Results

### (a) Spatial patterns of contemporary species richness and palaeoclimate changes

Species richness for the five taxonomic groups and 13 palaeoclimate estimates each demonstrates spatially structured patterns in eastern North America. Species richness is particularly high for amphibians, trees, and reptiles in the southeastern United States, while mammals have high species richness in the Appalachian Mountains, and birds exhibit more uniform richness across eastern North America (electronic supplementary material, figure S1). The well-documented phenomenon of greater amphibian, reptilian, and tree diversity in Florida and adjacent regions is captured in our richness estimates [25,154–156]. Differences in spatial configurations of richness among groups may be partly attributable to physiology—amphibians, trees, and reptiles are all ectotherms and should have greater richness in lower latitudes where ambient energy tends to be highest [157–160].

All climate models simulate little to no cooling across eastern North America south of 35° N during meltwater forcing events, and the southeastern United States emerges as a region of relatively warm and wet conditions amidst cooling and drying elsewhere (figure 1*a*). These spatially diverging climatological histories across eastern North America result in a steepening of the latitudinal temperature gradient across the Younger Dryas in proxy-based temperature estimates (figure 1*c*) [26] and a shallowing thereafter. Based on the ‘bipolar-seesaw’ conceptualization of interhemispheric temperature changes [161], the proxy data suggest a fulcrum to the latitudinal temperature anomalies positioned between 30 and 35° N, with little temperature change between 15 and 8 ka at these latitudes and larger changes further north (figure 1*c*). This palaeoclimate simulation-proxy agreement emerges as positive skill scores when quantitatively assessing climate model ability to capture the sign and magnitude of Younger Dryas temperature changes from the proxy reconstructions [27] (*x*-value in electronic supplementary material, figure S4). However, the spatial fingerprints of simulated temperatures differ in detail from the proxy data (figure 1*a*). Climate simulations on average indicate almost no temperature change across eastern North America and cooling in New England and maritime Canada (figure 1*a*). The spatial fingerprints of simulated precipitation changes are more consistent than simulated temperature changes, with all hosing simulations indicating increased precipitation in eastern North America concentrated on the coast near 38° N (figure 1*b*; electronic supplementary material, figures S14 and S15). This spatial pattern of simulated precipitation has mixed support in the proxy reconstructions, which indicate a more localized and heterogenous signal resulting in low skill scores [27] (*x*-value in electronic supplementary material, figure S5). The large and consistent simulated change to precipitation in the hosing-only climate simulations suggests that past millennial-scale variations in precipitation may exert their own legacies on contemporary biodiversity.

### (b) Trends and relationships between contemporary biodiversity and palaeoclimatic change

**Figure 2.**
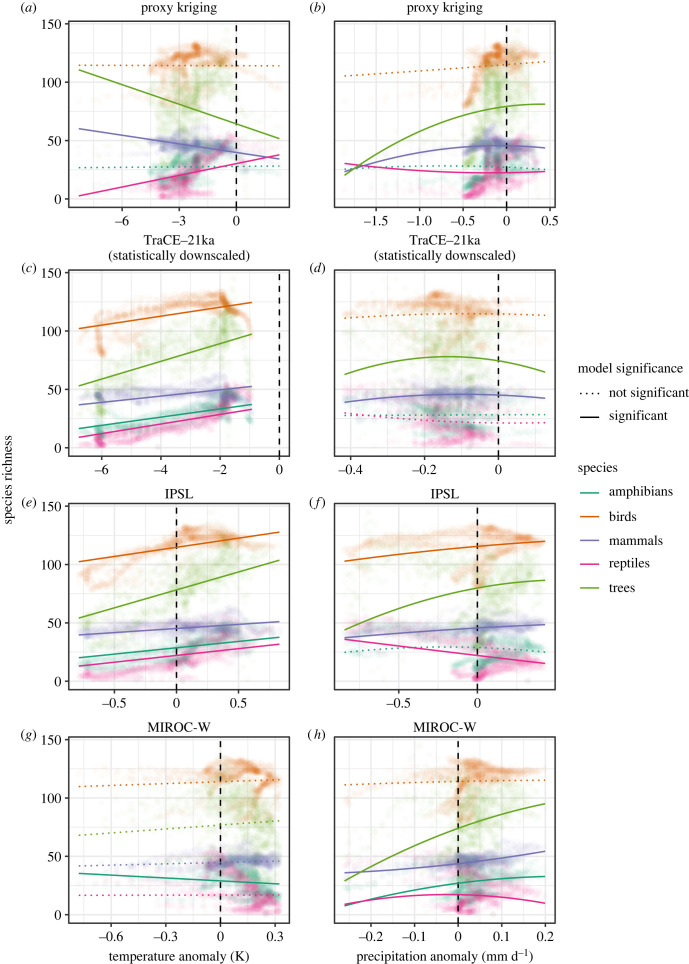
The modelled effect of millennial-scale climate change on contemporary species richness, based on hosed–unhosed anomalies for temperature and precipitation from the climate simulations and spatial error models (SEMs). Points correspond to species richness estimates for each taxonomic group paired with the corresponding grid cell climate anomaly. Under standard biogeographic theory, contemporary richness should be highest where past climate variability is lowest (i.e. anomalies are closest to zero which is marked by a black dashed line). To reduce figure complexity, a subset of the SEM results is shown, with climate models selected to show the range of patterns from alternative palaeoclimate sources and correlational outcomes. The full set of SEMs is available in the electronic supplementary material, figures S2 and S3, and individual SEM results are available in table S1. (*a,b*) SEMs where palaeoclimate predictors source from kriged fields of proxy-based temperature and precipitation reconstructions. (*b,c*) TraCE-21 ka (statistically downscaled by Lorenz *et al*. [61]) represent SEMs where the palaeoclimate predictors source from transient climate simulations. (*g,h*) IPSL represents SEMs where the palaeoclimate predictors source from hosing-only climate simulations and the relationship between palaeoclimate and contemporary species richness is significant. (*e,f*) MIROC-W represents SEMs with palaeoclimate predictors from hosing-only climate simulations but the relationship with contemporary richness is not significant. Note, palaeoprecipitation was fitted using a linear term and a quadratic term to account for nonlinearity in the relationship while palaeotemperature was fitted using only a linear term. For palaeoprecipitation a significant relationship was defined to mean that either the linear or quadratic terms were significant within the SEMs.

Millennial-scale temperature and precipitation changes emerge as weak to moderate predictors of the contemporary biodiversity of flora and fauna in eastern North America (figure 2). SEMs based on proxy-reconstructed temperatures generally show the weakest match to expectations; two taxonomic groups show no significant relationship (amphibians, passerine birds) and two others (trees, mammals) show the unexpected finding that regions with larger temperature decreases across the Younger Dryas have greater richness at present (figure 2*a*). This can probably be attributed to the kriging procedure and limited proxy data in the southeastern United States, which yield uncertain temperature estimates where data is sparse (figure 1; electronic supplementary material, figures S14 and S15). Modelled relationships tend to be closer to expectations for SEMs relying on palaeoclimate simulations. The SEM-modelled relationships for palaeotemperature generally agree that smaller millennial-scale temperature change is predictive of greater contemporary richness and support the hypothesis that the southeastern United States was a biodiversity refuge during the Younger Dryas (figure 2*c,g,h,f*). The apparent legacy effects are particularly strong for mammals, for which palaeoclimate correlates explain more deviance (i.e. improve SEM fit) in contemporary richness than modern temperature and precipitation (figure 2*c,e,f,h* and table 1). Trees, passerine birds, and reptiles also exhibit legacies of millennial-scale temperature changes, but contemporary mean annual temperature and precipitation are more predictive of richness than palaeoclimate correlates (figure 2 and table 1). This suggests that the southeastern United States may have acted as a refuge for these taxonomic groups, but this effect is weak, albeit significant. Amphibians demonstrate no legacies of millennial-scale temperature changes, because only six of the 10 hosing-only climate simulations are significantly predictive of contemporary richness and these relationships vary in direction (electronic supplementary material, figure S2). A lack of consensus among these alternate palaeoclimate estimates for amphibians suggests that millennial-scale temperature changes have either no effect on contemporary amphibian richness, or an effect too weak to overcome intermodel differences in palaeoclimatic simulations.

**Table 1.** Summary statistics from the SEMs, averaged by taxonomic group. (Per cent deviance explained is calculated as the average of individual SEMs, across 13 alternative palaeoclimate estimates used as predictors of richness. The last two columns correspond to the number of SEMs where modern climate predictors and palaeoclimate predictors were significant. For instance, millennial-scale temperature change was a significant predictor for amphibians in six of the 13 SEMs (see the electronic supplementary material, figure S2).

taxonomic class	modern climate (% deviance explained)	palaeoclimate (% deviance explained)	no. of SEMs with significant palaeoclimate correlates	no. of SEMs with significant modern climate correlates
amphibians	5.38	1.17	temperature 6/13	temperature 13/13
			precipitation 6/13	precipitation 6/13
birds	0.83	0.77	temperature 7/13	temperature 11/13
			precipitation 4/13	precipitation 1/13
mammals	0.88	1.52	temperature 8/13	temperature 10/13
			precipitation 8/13	precipitation 9/13
reptiles	10.21	1.91	temperature 9/13	temperature 13/13
			precipitation 8/13	precipitation 13/13
trees	3.22	1.32	temperature 9/13	temperature 13/13
			precipitation 7/13	precipitation 11/13

Palaeoprecipitation changes emerge as predictive for several taxonomic groups with legacies weaker than palaeotemperature, aligning with expectation (electronic supplementary material, table S1). Both proxies and hosing climate simulations suggest that positive palaeoprecipitation anomalies are predictive of greater contemporary richness for amphibians, trees, mammals and somewhat for passerine birds (figure 2*b,f,h*; table 1; electronic supplementary material, figure S3). TraCE-21ka and the statistically downscaled variant of TraCE-21ka were the only climate simulations that lack a linear relationship between richness and millennial-scale precipitation change for any taxonomic groups, although the relationship between past precipitation change and richness was U-shaped for mammals, trees and reptiles, centred on *ca* −0.1 mm d^−1^ (i.e. slight drying, figure 2*d*). Therefore, legacies of palaeoprecipitation may uniquely carry the influence of AMOC reduction because climate change resulting from AMOC reduction in TraCE-21ka is overprinted by changes in greenhouse gas concentrations and ice sheet topography [27]. Nevertheless, our expectation that species richness would be highest in regions with the smallest precipitation changes is not supported by the SEMs for nearly all palaeoclimate estimates.

Reptiles are the only taxon group with no clear relationship with millennial-scale precipitation change (figure 2). Although eight of the palaeoprecipitation estimates are significantly predictive of contemporary reptile richness, these relationships diverge (figure 2; electronic supplementary material, figure S3). Increases in palaeoprecipitation are predictive of both lower and higher contemporary richness, with upward and downward curvature, depending on the climate simulation used within the SEM (figure 2; electronic supplementary material, figure S3). Reptilian species richness is high along the southern coast of the southeastern United States (electronic supplementary material, figure S1), which is a region of model disagreement (electronic supplementary material figure S15). Climate simulations that indicate an increase in precipitation in this region yield a positive relationship between reptilian species richness and palaeoprecipitation (COSMOS-S, MIROC-S; electronic supplementary material, table S1 and figures S3 and S15). By contrast, IPSL and TraCE-MWF simulate drying along the southern coast and produce a negative relationship between reptilian species richness and palaeoprecipitation (electronic supplementary material, table S1 and figure S15). Hence, for reptiles, the intermodel uncertainties in coastal palaeoprecipitation are too high to assess hypotheses about past controls on present richness.

**Figure 3.**
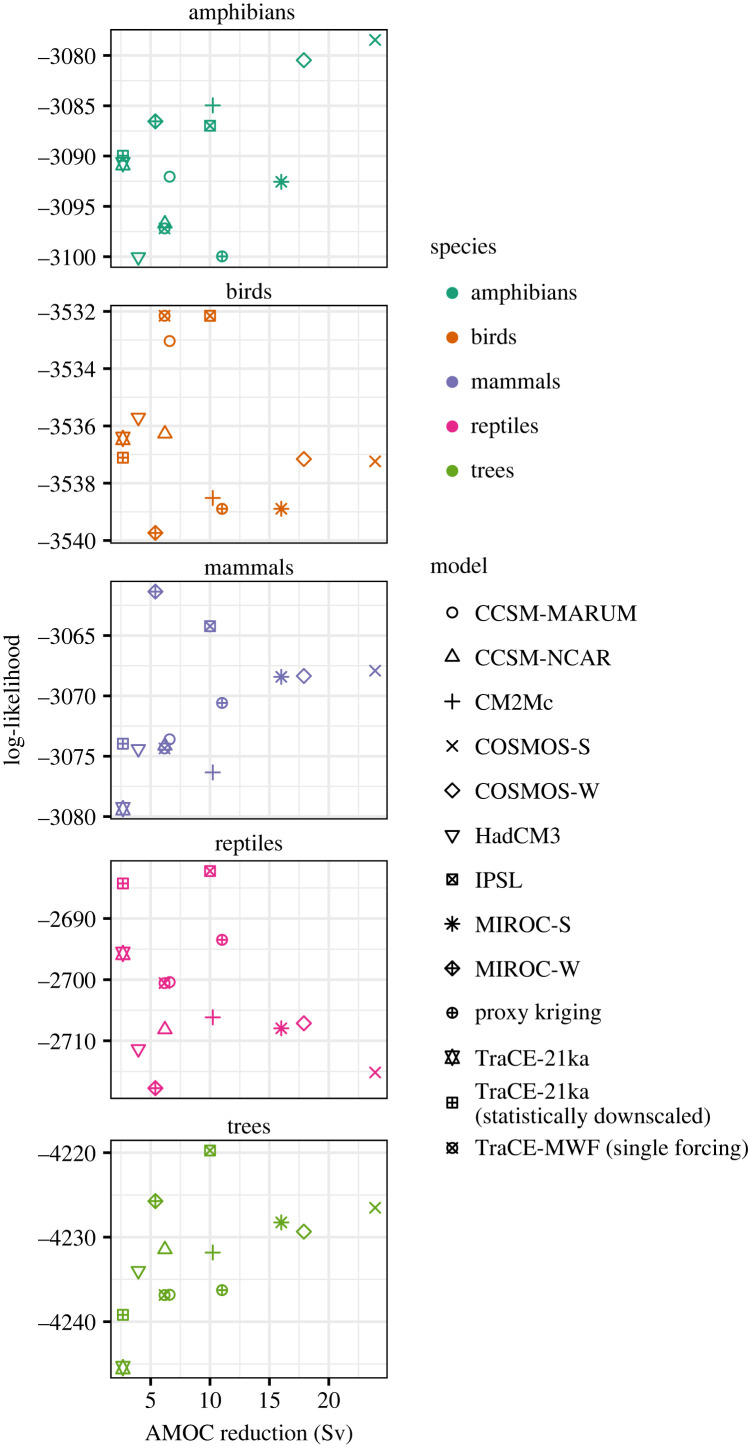
SEMs model fit measured by log-likelihood compared to the reduction in the strength of the Atlantic meridional overturning circulation (AMOC) in the climate simulations. The point symbology indicates the different sources of palaeoclimate estimates used within the SEMs, while the colours indicate taxonomic group, as in figure 2. Estimates of AMOC reduction in the proxy record are based on estimates from Ritz *et al*. [162] where AMOC strength was calculated from proxy based sea surface temperatures and climate simulations. Results suggest that, for some taxonomic groups (amphibians and trees), models with larger simulated reductions in AMOC may produce palaeoclimate simulations that better predict contemporary biodiversity.

Collectively, the SEMs suggest that the southeastern United States may have acted as a biodiversity refuge but the contemporary legacies of this are small relative to contemporary predictors. Modern climate predictors nearly always improve SEM fit more than palaeoclimate predictors (table 1). The improvements to model fit gained by adding palaeoclimate predictors in eastern North America are similar to, but somewhat lower than, those reported for Europe when using the same statistical framework [51]. A stronger relationship between millennial-scale climate variability and contemporary richness is expected in Europe, which is directly downwind of the north Atlantic and so was heavily exposed to past millennial-scale climate variability [9]. Nevertheless, the consensus among alternate palaeoclimate estimates in the direction of the palaeotemperature-richness relationships for mammals, and to a lesser extent trees, birds, and reptiles, provides evidence that the southeastern United States may have acted as a refuge from millennial-scale temperature oscillations. Similar consensus for palaeoprecipitation suggests that millennial-scale changes in precipitation may have influenced high contemporary biodiversity, but not as expected. Palaeoprecipitation increases were generally more predictive of contemporary richness, opposite to expectations. These weak legacies in the contemporary biodiversity of eastern North America suggest a connection between a reduction of the AMOC, climate changes in eastern North America and biodiversity. Except for TraCE-MWF, climate simulations with a greater reduction of the AMOC are the most predictive of amphibian, mammalian, and tree richness (figure 3).

## 5. Discussion

### (a) Legacies of millennial-scale climate change on contemporary biodiversity: implications

Our results indicate that past millennial-scale climate variations resulting from rapid changes to the AMOC, may exert weak but detectable legacies on contemporary patterns of biodiversity in eastern North America. Specifically, the high biodiversity of the southeastern United States may be partially explained by the limited amplitude of temperature variation and precipitation increases in this region during past hosing events. Disrupting the northward transport of heat by weakening the AMOC results in global reorganizations in temperature and precipitation patterns, which manifest in eastern North America as a cooling north of 35° N and a warming and wetting south of this latitude in the proxy record (figure 1). Biogeographic theory predicts that species richness and endemism are highest in areas of high past climate stability, because these regions allow persistence of species with high climate sensitivity and low dispersal capacity [81,87,163,164]. This effect of climate stability, combined with dispersal limitations on post-glacial range dynamics, has previously been used to explain legacies of past climatic changes for amphibians, reptiles, mammals, birds and trees but nearly all have focused predominantly on glacial-scale climate changes, comparing the Last Glacial Maximum to pre-industrial climates [3,6,51,86].

The strong effect of AMOC-driven climate variations on past ecological dynamics [22,165–167] and apparent legacy effects on contemporary biodiversity is notable, given that the AMOC is currently in the weakest state of the last millennium [168] and recent observations suggest a continuing weakening [169]. Simulations of future climate change forecast a 12–54% decrease in the strength of the AMOC under high-emission scenarios by 2100 [170] in response to meltwater and warming climates, which will substantially affect regional climates [171]. These shifts in AMOC are likely to have large effects on climates in and adjacent to the north Atlantic, and hence may be important for regional ecosystem trajectories. A key research need is to further refine the role of AMOC variations on past and present biodiversity using state-of-the-science climate models.

Our identification of climatic legacies in response to millennial-scale climate change builds upon prior research on whether rates of ecological response are fast or slow relative to past and present rates of climate change [172–176]. Estimates of rates of ecological response depend on the phenomenon studied, spatio-temporal scale, and measurement system. Fossil-pollen records generally indicate fast changes in plant abundances and community composition in response to the Younger Dryas [22]. However, fossil-pollen records usually capture changes in dominant plant taxa best [177–180], which may have quicker response times than rarer taxa. Range-filling analyses of contemporary tree distributions in eastern North America have suggested that trees and shrubs with small ranges are not in equilibrium with contemporary climate and may reflect dispersal limitations or other non-climatic controls on range limits [181]. New sedimentary ancient DNA records from northern Norway suggest a nearly continuous process of species immigration and richness accumulation over the last 10 000 years, suggesting strong legacy effects from the last deglaciation [182]. The strength, however, of glacial climate refugia and legacy effects may differ between eastern North America and Europe, as suggested by intercontinental differences in genetic diversity. Genetic legacies appear to be larger in Europe than in eastern North America, which generally has less of a latitudinal gradient in genetic diversity [183–186].

Our results also provide a more detailed regional-scale underpinning and mechanistic climate interpretation for global analyses which find that deglacial temperature and precipitation variations are predictive of contemporary biodiversity [1]. The lower millennial-scale climate variability in eastern North America compared with Europe is owing to its position upwind of the north Atlantic, which exposed most of Europe to abrupt cooling following a reduction of the AMOC [187,188]. During the Younger Dryas, the upwind position of eastern North America may have facilitated warming as atmospheric reorganization brought warm and moist air from lower latitudes into the southeastern United States [27]. Hence, the southeastern United States may have acted as a biodiversity refuge because of its fulcrum position adjacent to north Atlantic cooling and unique climate history of warming and wetting during millennial-scale climate oscillations [26,27,189]. Whereas recent work has called attention to the role of climatic dipoles in organizing macro-scale ecological phenomena such as tree masting and bird irruptions [190], our work suggests that the fulcrum regions adjacent to domains of high climate variability may be an under-appreciated mechanism for climate stability and preserving biodiversity. This is particularly relevant for millennial-scale climate oscillations, which are often attributed to a ‘bipolar-seesaw’ [161].

Lastly, while we focus on biogeographic implications of past millennial-scale climate variability, that variability may have also been important for early human history in North America. Models of forager populations in Europe during the most recent deglaciation suggest that population densities decreased during the Younger Dryas in response to temperature seasonality and energy availability [191]. It is possible that muted millennial-scale temperature variations and precipitation increases in the southeastern United States may have facilitated the population expansion of the earliest peoples in the Americas which inhabited the southeastern United States as early as 14 500 years before present [192].

### (b) Intermodel variance and implications for biodiversity modelling

Variability in our SEM results among palaeoclimate simulations underscores the importance of climate model selection for ecological and biogeographic interpretations. Although most palaeoclimate estimates agree that lower millennial-scale temperature anomalies are predictive of greater contemporary biodiversity, the strength and directionality of this relationship can vary, with MIROC-W as an endmember with no detectible relationship between millennial-scale temperature change and biodiversity for most taxonomic groups (figure 2). The sensitivity of ecoclimate interpretations to climate model choice is particularly high for reptiles: depending on the climate simulation used within the SEM, either reduced or increased precipitation anomalies can predict greater reptilian richness.

Accordingly, we urge caution when interpreting ecological relationships from individual climate models, especially at regional scales. Incorporating multiple climate models, such as the ensembles provided by the Climate Model Intercomparison Project [193] and the Paleoclimate Modelling Intercomparison Project [194,195], is common throughout climatological and palaeoclimatological analyses (e.g. [196–200]) and is becoming more common in ecological research (e.g. [201–203]). The palaeoclimate simulations downscaled in WorldClim v1.4 [204] are widely used in biogeography. A next generation of downscaled palaeoclimate estimates based on all Paleoclimate Modelling Intercomparison Project 4 simulations [205] would enable easily incorporating state-of-the-science palaeoclimate simulation ensembles within biogeographic models. This generation of multi-member palaeoclimatic ensembles should be accompanied by open-source software that allows end-users to easily access and post-process climate simulation output from the Paleoclimate Modelling Intercomparison Project and the Coupled Model Intercomparison Project.

For transient simulations, which are computationally expensive and hence rarer, incorporating an ensemble of climate simulations within biogeographic analyses is more challenging. Few transient simulations of the most recent deglacial climate exist with TraCE-21ka being the standard in palaeoclimatic, palaeoecological and contemporary ecological research [58,59]. Yet, even the TraCE-21ka simulations have known discrepancies with palaeoclimatic data. For instance, the meltwater forcing is larger than proxy-constrained estimates [113] and the timing of greenhouse gas forcings for the Bølling-Allerød and Younger Dryas is delayed by about 600 years [63,64], which adds uncertainty to the climate simulations. Seasonal precipitation simulations are particularly challenging: in North America TraCE-21ka simulations appear to be the most accurate where summer precipitation is dominant, and least accurate where winter precipitation predominates [32]. At subregional scales in eastern North America, simulated and reconstructed fingerprints of precipitation change show discrepancies, perhaps because of the coarse spatial resolution (*ca* 3°) of the TraCE-21ka simulations [27]. An exciting advancement is the upcoming Paleoclimate Modelling Intercomparison Project and its deglacial transient simulations [206], which will enable checking of new ecoclimate findings against earlier interpretations based on the TraCE-21ka simulations.

## 6. Conclusion

We examine the legacies of past millennial-scale climate change in the contemporary distribution of amphibians, passerine birds, mammals, reptiles and trees in eastern North America. We demonstrate that past millennial-scale variations in the strength of the AMOC may have influenced contemporary patterns of species richness but weakly. The relationship between past climate changes and contemporary species richness varies depending on the source of palaeoclimatic estimates and the taxonomic group analysed but patterns emerge. Of the 13 palaeoclimate estimates, nearly all indicate that lower-amplitude variations in temperature and increases in precipitation across a millennial-scale climate event predict greater contemporary biodiversity for all taxonomic groups except amphibians and reptiles. This pattern is particularly strong for mammals, and to a lesser degree for trees. The low-amplitude climate variations in the southeastern United States during millennial-scale climate variations may explain why this region has acted as a refuge for biodiversity. An important secondary finding is that variations in the strength and direction of this relationship depends on the source palaeoclimate estimate used within the SEMs. Considering this sensitivity of biogeographic modelling to data source, we urge caution when making ecological interpretations based on analyses of a single palaeoclimate simulation.

## Data Availability

Code and data for analysis are available from the Zenodo repository: https://doi.org/10.5281/zenodo.7893779 [207]. Climate simulation data is available upon request from the corresponding author. Data is also available in the electronic supplementary material [208].
